# Determinants of growth differentiation factor 15 plasma levels in outpatients with peripheral arterial disease

**DOI:** 10.48101/ujms.v129.11001

**Published:** 2024-12-23

**Authors:** Emma Skau, Philippe Wagner, Jerzy Leppert, Johan Ärnlöv, Pär Hedberg

**Affiliations:** a Centre for Clinical Research, Uppsala University, Västmanland County Hospital, Västerås, Sweden; b Department of Cardiology, Danderyd University Hospital, Stockholm, Sweden; c School of Health and Social Studies, Dalarna University, Falun, Sweden; d Division of Family Medicine and Primary Care, Department of Neurobiology, Care Sciences and Society (NVS), Karolinska Institutet, Huddinge, Sweden; e Department of Clinical Physiology, Västmanland County Hospital, Västerås, Sweden

**Keywords:** Atherosclerosis, biomarker, GDF-15, peripheral arterial disease, diabetes

## Abstract

**Background:**

Growth differentiation factor 15 (GDF-15) is a robust prognostic biomarker in patients with cardiovascular (CV) disease, and a better understanding of its clinical determinants is desirable. We aimed to study the associations between GDF-15 levels and *traditional CV risk factors, indicators of atherosclerotic burden, and cardiac geometry and dysfunction* in outpatients with peripheral arterial disease (PAD).

**Methods:**

An explorative cross-sectional study (Study of Atherosclerosis in Vastmanland, Västerås, Sweden) included 439 outpatients with carotid or lower extremity PAD. The mean age was 70 years (standard deviation [SD] 7), and 59% of the patients were men. Plasma levels of GDF-15 were obtained along with potential determinants, including medical history, biochemical data, echocardiographic measures of cardiac geometry and function, ankle-brachial index (ABI), and carotid ultrasonographic data on intima-media thickness (IMT) and occurrence of carotid stenosis. The relations between GDF-15 concentrations (transformed with the natural logarithm) and the different determinants were evaluated using uni- and multivariable linear regression models. All pre-specified variables were included in the multivariable models.

**Results:**

The multivariable analysis identified independent relations of GDF-15 with several of the included variables (adjusted *R*^2^ = 0.48). Diabetes (beta coefficient [β] of 0.37, 95% confidence interval [95% CI] 0.25 to 0.50), low-density lipoprotein (LDL) cholesterol (β = −0.22, 95% confidence interval [CI]: −0.34 to −0.09), and physical activity (β = −0.16, 95% CI: −0.25 to −0.06) had the strongest associations. In contrast, no significant independent associations with GDF-15 level were observed for cardiac geometry and function, ABI, IMT, or carotid stenosis.

**Conclusions:**

Circulating GDF-15 is more strongly associated with traditional CV risk factors, especially diabetes, LDL cholesterol, and physical activity than with specific indicators of atherosclerotic burden or cardiac dysfunction. To better understand the pathophysiological role of GDF-15 and its link to clinical outcomes in patients with PAD, future studies should focus on the metabolic processes involved in atherosclerotic disease.

## Introduction

Growth-differentiation factor 15 (GDF-15), also named macrophage inhibitory cytokine-1, is a member of the transforming growth factor β superfamily (1). It is a strong and independent predictor of mortality and disease progression in patients with various cardiovascular (CV) diseases (2–4) and other diseases such as cancer (5). The pathophysiological mechanisms linking this biomarker to the outcomes have not yet been clarified. GDF-15 appears to exhibit different functions in various contexts, including pro- and anti-apoptotic, pro- and anti-angiogenic, and pro- and anti-inflammatory properties (6). Studying the association of GDF-15 plasma levels with different determinants among individuals with atherosclerotic diseases may provide clues to clarify its role in atherosclerotic pathophysiological paths, and thereby, a better understanding of the prognostic impact in these patients. Such studies on patients with established peripheral arterial disease (PAD) are scarce (7).

In the present exploratory study, we aimed to investigate the relationship between GDF-15 and traditional CV risk factors, cardiac geometry and function, and atherosclerotic burden in outpatients with PAD of the carotid and lower limb arteries.

## Method

The current study is a cross-sectional analysis investigating the relationship of GDF-15 with traditional risk factors and humoral biomarkers, indicators of atherosclerotic burden and cardiac geometry and function in outpatients with PAD. The purpose of this study is to gain a deeper understanding of the role of GDF-15 in the atherosclerotic process.

### Study population

The Peripheral Artery Disease in Västmanland study (PADVa) included consecutive patients referred to the Vascular Ultrasound Laboratory of the Department of Vascular Surgery, Västmanland County Hospital, Västerås, Sweden, between April 2006 and February 2011. The population has been previously described in detail (8). The prognostic value of GDF-15 has been previously reported in this population (4). Reasons for referral included intermittent claudication (45%), transient ischemic attack or stroke (26%), aortic aneurysm (8%), heart murmur (5%), suspected renal artery stenosis/renovascular hypertension (4%), and others (12%). All patients underwent an ultrasound examination of the carotid arteries. Patients reporting intermittent claudication symptoms (described as lower limb pain reproduced by exercise and relieved within 10 min rest) underwent ankle blood pressure measurement and duplex ultrasonographic evaluation of the arteries in the ipsilateral limb. No objective tests were performed, such as treadmill testing. At least one of the following criteria was required to be met to get enrolled in this study:

- ultrasound verified stenosis or occlusion of the internal carotid artery (ICA),
- symptoms of intermittent claudication with ankle-brachial index (ABI) ≤0.9 or
- symptoms of intermittent claudication with signs of occlusive arterial disease on ultrasound examination in the ipsilateral extremity.

Among 614 participants fulfilling the criteria for inclusion, 162 (26.4%) rejected inclusion, and 13 (2.1%) had missing values of GDF-15, leaving 439 for inclusion.

The Regional Ethical Review Board in Uppsala, Sweden, approved the study (Dnr 2005:382 and 2005:382:2). Written informed consent was obtained from all participants.

### Study protocol

We collected information on smoking habits, physical activity, medical history, and medication from self-administered questionnaires. Self-reported prevalent medical diagnoses of diabetes and previous CV diseases were confirmed from medical records. Hypertension was considered present if diagnosed by a physician and pharmacologically treated. Smoking was identified based on self-reported information and was defined as previous or current smoking. The subjects were asked to describe their current leisure-time activity level as ‘low’ (mostly sedentary with more demanding activities such as walking, biking, or gardening <2 h per week), ‘moderate’ (primarily sedentary, but more demanding activities such as walking, biking, or gardening ≥2 h per week, usually without breaking a sweat), ‘high’ (vigorous exercise with sweating for at least 30 min, ≥ 1–2 times per week).

### Ankle-brachial index and vascular ultrasound

The ABI was calculated as the highest systolic blood pressure in the dorsalis pedis artery and the posterior tibial artery divided by the systolic blood pressure in the arm. ABI was defined as abnormal if ≤0.90 or ≥1.40 in any leg.

Ultrasound examinations of the internal carotid and lower limb arteries were performed with convex or linear 4–8 MHz probes on Acuson Sequoia systems (Siemens Healthcare GmbH, Erlangen, Germany). Stenosis of the ICA was considered when a localized protrusion of the vessel wall into the lumen in combination with turbulent color Doppler flow, spectral broadening in the spectral Doppler flow, and a peak systolic flow velocity of 1.2–1.4 m/s (mild stenosis), 1.2–2.5 m/s (moderate stenosis), or ≥2.5 m/s (severe stenosis) was detected. Occlusion was considered if no Doppler flow was detected in the presence of a localized atherosclerotic plaque.

Stenosis in the lower extremity arteries was considered in case of a turbulent color Doppler flow, widening of spectral Doppler flow, lack of reversed flow distal to the stenotic segment, and an increase of peak flow velocity to >150% (iliac arteries) or >100% (arteries below the iliac arteries). Occlusion was assumed when no flow over a localized segment in combination with a low peak flow velocity with a monophasic flow profile distal to the segment was detected.

Images to assess carotid intima-media thickness (IMT) were acquired at the time of the echocardiographic examinations using an 8 MHz linear transducer (Vivid 7, GE VingMed Ultrasound, Horten, Norway). A semi-automated software (Echopac, GE VingMed Ultrasound, Horten, Norway) was used to measure IMT in the common carotid artery (CCA) (9). The operator placed a 1 cm long region-of-interest (ROI) over the far wall of the CCA, 1–2 cm proximal to the carotid bulb, in an image frame frozen in cardiac end-diastole. The CCA’s lumen–intima and media–adventitia interfaces were then automatically detected by the software algorithm within the ROI. An average IMT was calculated from approximately 250 data points. The average of six such IMT measurements (three from the left and three from the right CCA) were used for analysis.

### Echocardiography

The echocardiographic examinations were performed using Vivid 7 systems (GE Vingmed Ultrasound, Horten, Norway). The left ventricular (LV) dimensions and mass and left atrial (LA) volumes were measured and calculated according to the European Association of Echocardiography (10). The LV mass and LA volumes were indexed by dividing with the body surface area. The LV ejection fraction (LVEF) was evaluated using the biplane Simpson’s formula.

### Biochemistry

All the blood samples were taken after a night of fasting and sent to the accredited Laboratory of Clinical Chemistry, Västmanland County Hospital, Västerås, Sweden, for analyses or freezing. Serum creatinine and low-density lipoprotein (LDL) cholesterol levels were measured enzymatically using Synchron LX or UniCel DxC instruments (Beckman Coulter, USA). The estimated glomerular filtration rate (eGFR) was calculated using the CKD-EPI formula (11).

HbA1c was measured with high-performance liquid chromatography using cation exchange separation and calibrated against the Swedish Mono S method (TOSOH automated Glycohemoglobin Analyzer G7, Tosoh, Japan). HbA1c Mono S was converted to IFCC units using the equation *HbA*1*c* (*IFCC*) = *HbA*1*c* (*mono S*) × 10.45–10.62. This formula differs slightly from the IFCC master equation *HbA*1*c* (*IFCC*) = *HbA*1*c* (*mono S*) × 10.11–8.94. and is due to a recalculation in 2004 (12, 13).

Blood samples for highly sensitive C-reactive protein (hs-CRP) and GDF-15 analysis were obtained in 5 mL lithium heparin-coated vacuum tubes. The tubes were centrifuged at 2,000 g for 10 min (Becton Dickinson and Co., Franklin Lakes, New Jersey, USA) or 2,200 g for 10 min (Vacuette, Greiner Bio-One GmbH, Austria). The plasma was reallocated to 5 mL plastic tubes, frozen, and stored at −70°C within 2 h. Before analysis, the samples were thawed at room temperature, mixed, centrifuged at 3,470 g at 4°C for 15 min, and aliquoted into a microtiter plate using a pipetting robot, the Tecan Freedom Evolyze. The analyses were performed in 2017 at the Clinical Biomarkers Facility, Science for Life Laboratory, Uppsala University, Uppsala, Sweden. Hs-CRP plasma levels were analyzed with chemiluminescence immunoassay on the Cobas Analytics e501 (Roche Diagnostics, Mannheim, Germany). Plasma levels of GDF-15 were analyzed by an immunoassay based on the specific Elecsys chemiluminescence (ECL) detection system (Roche Diagnostics, Mannheim, Germany) (14). The analytical range was 400–20,000 ng/L with a total analytical imprecision (coefficient of variation) of 4.9%.

### Statistics

We present continuous variables as mean and standard deviation (SD) or median and interquartile range. Categorical variables are presented as frequency and percentage. The distribution of GDF-15 in the full study population and subgroups is visualized using density plots.

Uni- and multivariable linear regression modeling was performed to identify the associations between the concentrations of GDF-15 and contemporary recorded risk factors, indicators of atherosclerotic burden, and variables of cardiac geometry and function. The estimates of the linear regression models are presented as beta coefficients and 95% confidence intervals. For categorical independent variables, the beta coefficients correspond to the difference between category levels, whereas for continuous variables, it corresponds to one SD increase in the independent variable. Variables included in the multivariable model were sex, age, smoking, body mass index (BMI), hypertension, diabetes, previous myocardial infarction (MI), previous stroke, physical activity, systolic blood pressure, eGFR, LDL cholesterol, hs-CRP, HbA1c, abnormal ABI, IMT, ICA stenosis, LV mass index, LVEF, and LA volume index. Due to highly skewed distributions, GDF-15, HbA1c, and hs-CRP were transformed with the natural logarithm. In the multivariable model, all prespecified variables were included. All continuous variables were plotted against log-GDF-15 in scatterplots.

We used restricted cubic splines (RCS) with three knots at the independent variables’ 10th, 50th, and 90th percentiles to evaluate potential nonlinearity in these associations. A Wald test was used for the coefficients of the spline terms only to test the null hypothesis that the association was linear. The null hypothesis could be rejected for eGFR and LDL cholesterol; therefore, these variables were included in the regression models using the RCSs.

Missing values were imputed by multiple imputation by chained equations (MICE) (15). The missing values are presented in Table 1. The assumption made for the missing data mechanism was Missing at Random (MAR), meaning that the probability of a value being missing depends on the observed data but not on the missing values themselves. This assumption allows the imputation model to use available observed information to impute missing values. We chose a number of imputed datasets (*M* = 50) based on the fraction of incomplete cases (FIC = 0.337), that is above the recommended ≥ FIC × 100. The imputation model included all the variables prespecified for the final multivariable linear regression model. The MICE procedure was iterated 30 times before imputation. Parameter estimates from analyzing the imputed datasets were pooled according to Rubin’s rules (16). Sensitivity analyses were performed by increasing and decreasing the imputed values of LVEF and IMT (the two variables with the most prevalent missing values) separately by 20% in the linear regression models to assess the robustness of our results to potential deviations from the MAR assumption. In addition, we performed a complete case analysis (i.e. excluding all patients with missing values).

**Table 1:** Characteristics of 439 outpatients with peripheral arterial disease according to tertiles of GDF-15.

Variables	All patients	Missing values	1^st^ Tertile (<1,108 ng/L)	2^nd^ Tertile (1,108–1,762 ng/L)	3^rd^ Tertile (>1,762 ng/L)
Patients, *n*	439		147	146	146
**Clinical variables**					
Age (years)	70.0 (± 7.3)		67 (± 7.5)	70.9 (± 6.4)	72.1 (± 6.8)
Male	260 (59.2)		74 (50.3)	84 (57.5)	102 (69.9)
Smoking	332 (75.8)	1	107 (72.8)	106 (72.6)	119 (81.5)
BMI (kg/m^2^)	27.1 (± 5.2)		27.0 (± 4.07)	27.3 (± 4.2)	27.1 (± 4.3)
Hypertension	338 (77)		110 (74.8)	105 (71.9)	123 (84.2)
Diabetes	110 (25.1)		17 (11.6)	25 (17.1)	68 (46.6)
Previous MI	80 (18.2)		17 (11.6)	22 (15.1)	41 (28.1)
Previous stroke	44 (10.0)		9 (6.12)	16 (11.0)	19 (13.0)
Physical activity		1			
Low	25 (17.0)		25 (17.0)	38 (26.0)	51 (34.9)
Moderate	82 (55.8)		82 (55.8)	74 (50.7)	75 (51.4)
High	40 (27.2)		40 (27.2)	34 (23.3)	20 (13.7)
Systolic BP (mmHg)	145.0 (± 21.4)		146 (± 21.1)	147 (± 22.1)	144 (± 21.0)
**Biochemical variables**					
eGFR (mL/min/1.73 m^2^)	74.1 (± 20.4)		81.2 (1± 7.4)	76.3 (1± 8.9)	64.7 (± 21.3)
LDL cholesterol (mmol/L)	2.7 (± 0.9)	7	2.7 (± 0.9)	2.8 (± 0.10)	2.5 (± 0.9)
hs-CRP (mg/L)	4.7 (± 14.3)		2.9 (± 5.7)	3.0 (± 3.1)	8.0 (± 23.7)
HbA1c (mmol/mol)	42.5 (± 10.7)	1	39.5 (± 7.8)	41.1 (± 10.0)	46.9 (± 12.3)
**Peripheral atherosclerotic burden**					
Abnormal ABI	256 (58.3)		61 (41.5)	96 (65.8)	99 (67.8)
IMT (mm)	0.9 (± 0.2)	64	0.9 (± 0.2)	0.9 (± 0.2)	1.0 (± 0.2)
ICA Stenosis	330 (75.2)		111 (75.5)	98 (67.1)	121 (82.9)
**Cardiac geometry & function**					
LV mass index (g/m^2^)	105.0 (± 27.3)	5	98.7 (± 23.4)	103.0 (± 25.5)	112.0 (± 30.9)
LVEF (%)	60.9 (± 9.6)	87	62.5 (± 7.7)	60.8 (± 9.5)	59.3 (± 11.1)
LA volume index (mL/m^2^)	34.1 (± 11.6)	26	32.6 (± 9.4)	32.5 (± 9.6)	37.4 (± 14.5)

Values are mean ± standard deviation or frequency (percentage).

ABI: ankle-brachial index; BMI: body mass index; BP: blood pressure; eGFR: estimated glomerular filtration rate; GDF-15: growth differentiation factor 15; HbA1c: glycated hemoglobin; hs-CRP: high-sensitive C-reaction protein; ICA: internal carotid artery; IMT: intima-media thickness; LA: left atrial; LDL: low-density-lipoprotein cholesterol; LV: left ventricular; LVEF: LV ejection fraction; MI: myocardial infarction.

When controlling for multicollinearity by assessing variance inflation factors (VIF), no independent variable had a VIF value over 3.

Two-sided *P*-values <0.05 were considered statistically significant. R (R Foundation for Statistical Computing; 2016, Vienna, Austria; https://www.r-project.org) were used for the statistical analyses.

## Results

The characteristics of the study population are summarized in Table 1. The majority (75%) of the patients had ICA stenosis of at least mild grade, and 58% had an abnormal ABI. A combination of ICA stenosis and abnormal ABI was found in 35% of the patients. Among the 112 patients referred for TIA/stroke, 110 (98%) had at least mild grade, whereas 25 (22%) had severe ICA stenosis. The median self-reported distance that patients with intermittent claudication could walk before stopping was 150 m (interquartile range: 85–300 m). Lower limb ultrasound examination revealed stenosis or occlusion in the iliac, femoral, and popliteal arteries and in the arteries below the knee in 18, 58, 14, and 18% of patients referred for intermittent claudication, respectively.

**Figure 1:**
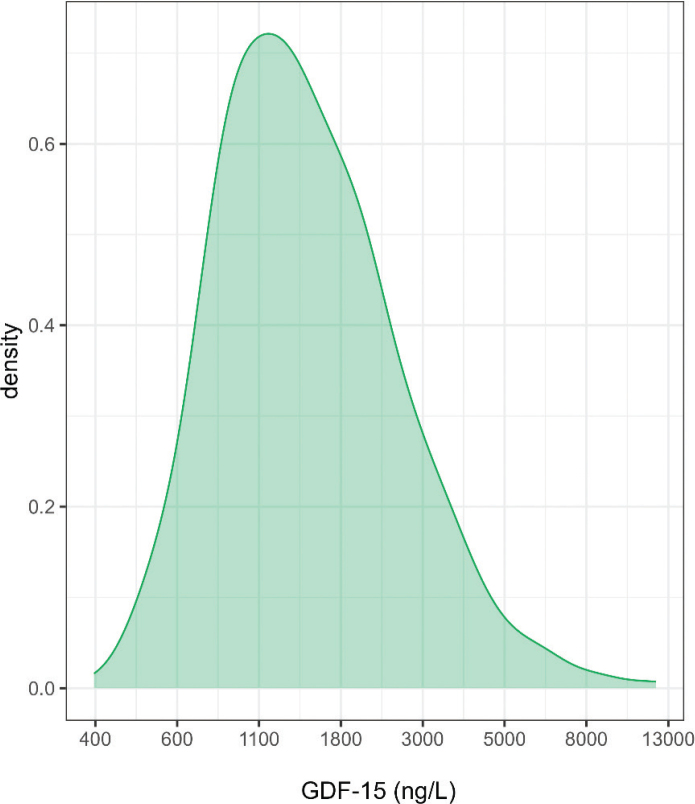
Density plot showing the distribution of GDF-15 in the study population. The x-axis represents a logarithmic scale.

GDF-15 levels in the study cohort ranged from 400 to 12,404 ng/L with a median (interquartile range) of 1,379 (978–2,081) ng/L. Figure 1 shows the distribution of the GDF-15 levels in the study population. The distribution of GDF-15 among subgroups is presented in Supplementary Figures 1 and 2.

### GDF-15 in relation to clinical and biochemical risk factors

LDL cholesterol and eGFR showed non-linear relations with GDF-15 and were, therefore, included in the models with RCS. Scatter plots with these variables against GDF-15 are shown in Supplementary Figures 3 and 4.

**Figure 2:**
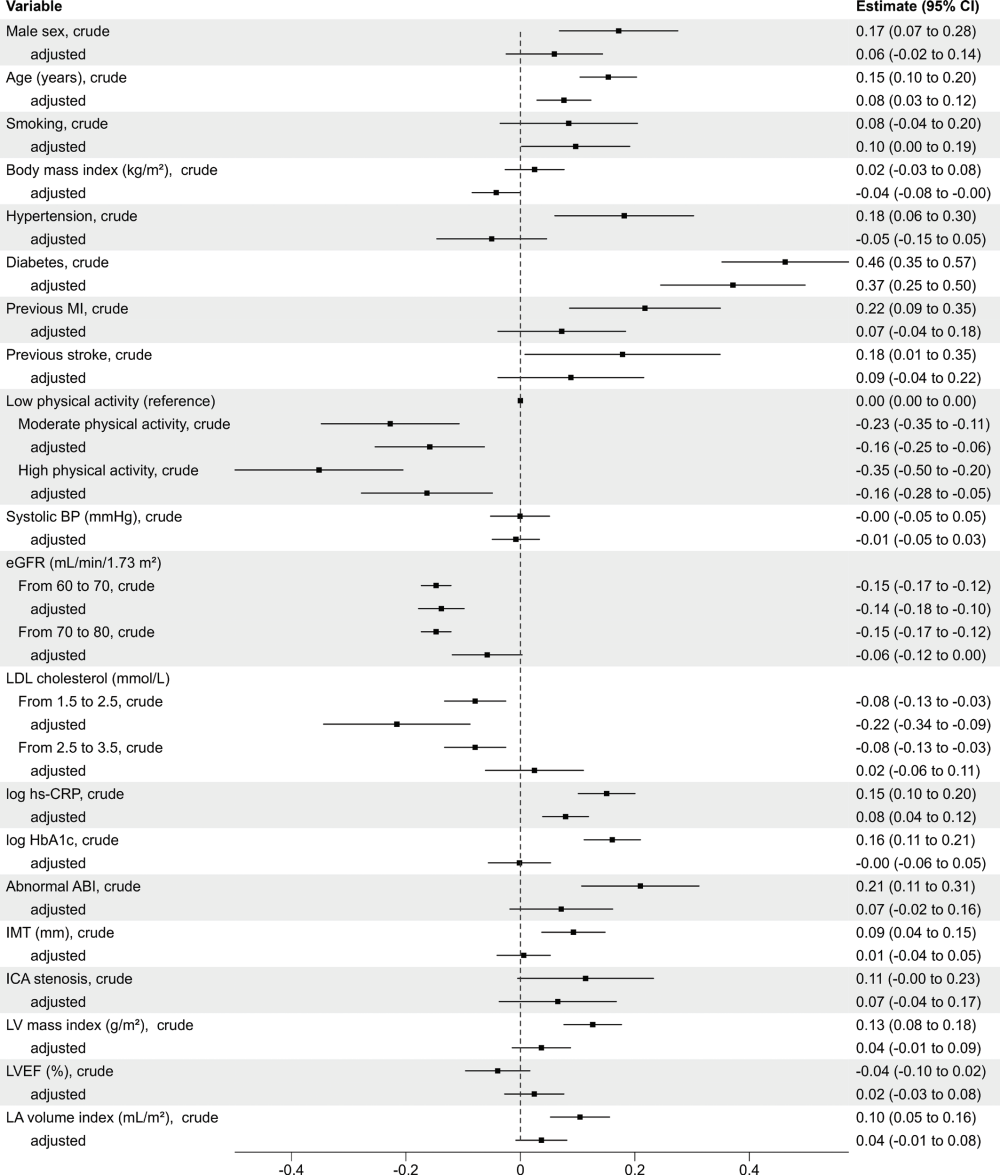
Forest plot showing associations of the natural logarithm of GDF-15 plasma levels with variables for clinical and biochemical risk factors, peripheral atherosclerotic burden, and cardiac geometry and function. The estimates are beta coefficients and 95% CI for every standard deviation increase in continuous variables and presence versus absence for categorical variables. For every variable, the first coefficient is unadjusted, and the second is adjusted for the other variables presented. ABI: ankle-brachial index; BP: blood pressure; CI: confidence intervals; eGFR: estimated glomerular filtration rate; GDF-15: growth differentiation factor 15; HbA1c: glycated haemoglobin; hs-CRP: high-sensitive C-reaction protein; ICA: internal carotid artery; IMT: intima-media thickness; LA: left atrial; LDL: low-density-lipoprotein cholesterol; LV: left ventricular; LVEF: LV ejection fraction; MI: myocardial infarction.

As shown in Figure 2, GDF-15 levels were associated with several variables in the univariable models. However, only age, smoking, diabetes, low physical activity, low eGFR, low LDL cholesterol, and high hs-CRP remained significantly associated with higher GDF-15 levels in the multivariable regression analysis. Diabetes and low LDL cholesterol had strong associations with beta coefficients of 0.37 (confidence interval [CI]: 0.25−0.50) and −0.22 (CI: −0.34 to −0.09), respectively. The multivariable model, with all prespecified variables included, explained 48% of the variance in GDF-15 levels (adjusted *R*^2^ = 0.48).

### GDF-15 in relation to peripheral atherosclerotic burden and cardiac geometry and function

In the univariable models, increased levels of GDF-15 were associated with abnormal ABI, IMT, greater LV mass index, and LA volume index. However, no significant associations were seen with ICA stenosis or LVEF. In the multivariate analysis, no significant associations existed to any variables reflecting atherosclerotic burden and cardiac geometry and function.

Sensitivity analyses using complete cases (Supplementary Figure 5) and shifting the imputed values for LVEF and IMT (Supplementary Figures 6–9) yielded similar results.

## Discussion

In this exploratory study, we investigated the association of plasma levels of GDF-15 with traditional risk factors and indicators of atherosclerotic burden and cardiac dysfunction among outpatients with manifest PAD in carotid and lower limb arteries.

The median level of GDF-15 among our subjects was higher than observed in a general population of the same age but lower than in a population with Acute coronary syndrome (ACS), heart failure, and also among subjects with severe lower limb ischemia (2, 7, 17, 18). This might reflect differences in age and disease burden among the populations studied.

### GDF-15 and traditional risk factors

Our results confirm findings in previous studies targeting populations with heart diseases and community-based populations, where higher GDF-15 has been independently associated with several clinical risk factors, such as higher age, smoking, diabetes, low physical activity, low eGFR, low LDL cholesterol, and higher hs-CRP (2, 4, 17, 19, 20).

In accordance with other studies on patients with MI, severe lower limb ischemia, and in a community-based population, we identified diabetes and low LDL cholesterol strongly associated with GDF-15 (2, 7, 17). GDF-15 has previously been demonstrated to be associated with future incident diabetes and to increase insulin sensitivity (21, 22). Metformin, an insulin-sensitizing agent, has been shown to increase the circulating levels of GDF-15 and mediate some of its effects (23, 24). That diabetes has been shown to have the strongest correlation to GDF-15 among other prognostic factors in a population with manifest PAD indicates that GDF-15 is linked to both diabetes and atherosclerosis and seems to be involved in the pathophysiology of both these entities. Previous studies have shown that increased GDF-15 levels protect endothelial cells against high glucose-induced cellular injury (25), but the exact pathophysiological role of GDF-15 behind this remains unclear.

Our results included a non-linear correlation between GDF-15 and LDL cholesterol. Lower LDL cholesterol levels were associated with higher levels of GDF-15, although this effect leveled off at LDL cholesterol concentrations of >2.5 mmol/L. The inverted correlation of low LDL cholesterol with GDF-15 levels corroborates previous findings (17). The oxidative form of LDL cholesterol is essential in atherosclerotic pathophysiology (26). It has been suggested that GDF-15 is induced by oxidized LDL cholesterol in macrophages located in human atherosclerotic carotid arteries (27). How this pathophysiological link correlates with low LDL cholesterol levels and the exact roles of GDF-15 in this context need to be further explored.

It is known that very low cholesterol is a biological marker for concurrent cachexia, malnutrition, cancer, and other chronic diseases with proven adverse impacts on survival (28, 29). GDF-15 is involved in energy homeostasis and body weight regulation and plays a distinct role in cachexia (30). This connection might explain the association between shallow LDL cholesterol levels and GDF-15 in patients with PAD, which needs further exploration.

It is well-known that inflammation has a crucial role in the development and progression of atherosclerosis (31). CRP appears to induce GDF-15 expression through the regulation of p53, which leads to an inflammatory response and vascular injury (32). In elderly populations and among patients with heart disease and carotid atherosclerosis, GDF-15 levels are related to circulating markers of inflammatory activity as CRP (2, 17, 33). GDF-15 is produced in activated macrophages (34), and it has been suggested that GDF-15 has a central role in the atherosclerotic process through pro- and anti-inflammatory qualities (6).

Renal failure is associated with atherosclerosis, and eGFR is a robust independent predictor of CV outcomes (35). Elevated GDF-15 concentrations have been associated with a greater decline in eGFR, renal disease progression, and incidence of chronic kidney disease (CKD) (36, 37). Persistent, low-grade inflammation is recognized as an important component of low eGFR and CKD (38).

GDF-15 levels tend to increase as people age, and they are usually low in individuals who are healthy and young (19, 39). GDF-15 levels dramatically increase in chronic or acute illnesses and age-related diseases (5, 6, 39). Inflammation is claimed to be a key pathophysiological mechanism in aging. Our results suggest that inflammation might be a common denominator in these relations to the abovementioned clinical and biochemical risk factors. Whether GDF-15 is a mediator in inflammation and atherosclerosis or a response to inflammatory processes involved in vascular injury must be clarified.

Smoking was associated with GDF-15 in this study, although the association was not strong. Smoking generates inflammatory processes (40), which might be an explanation for its link to high levels of GDF-15.

We found a strong association between physical activity and low GDF-15 levels. There is a well-known health benefit of physical activity (41). Physical activity among older people and higher levels of cardiorespiratory fitness among 50-year-old individuals has been associated with lower levels of GDF-15 (42).

### GDF-15 and atherosclerotic burden

The variables of atherosclerotic burden, such as ABI, IMT, and ICA stenosis, did not show any significant independent associations with GDF-15. In the univariable analysis, GDF-15 was associated with ABI and IMT, but this association was attenuated after adjustment. This finding could be due to insufficient power in our study to detect associations or to the absence of any association with these variables. IMT and ABI are markers for the presence and progression of atherosclerosis and strong predictors for future CV events (43–45). A previous study demonstrated associations with GDF-15 and variables indicative of vascular dysfunction in a general population (17). As in our study, there was no significant association between GDF-15 and ICA IMT after adjustment. However, after adjustment, other variables, such as a reduced capacity for endothelium-dependent vasodilation in resistance vessels, remained significantly associated with GDF-15. Atherosclerotic mouse models have shown a causal role of GDF15 in the chemotaxis of macrophages to the plaque, specifically during the early phase of atherosclerosis (46). In a previous study involving patients at an advanced stage of atherosclerosis, GDF-15 did not relate to plaque characteristics such as macrophage amount, calcification, intraplaque fat, and hemorrhage (7). GDF-15 might be more actively involved in the early phase of an atherosclerotic process. This could explain why GDF-15 had no significant correlation to indicators of atherosclerotic burden in patients with manifest PAD compared to a general population with a lower atherosclerotic disease burden.

### GDF-15 and cardiac geometry and function

We found an association between GDF-15 and variables of cardiac geometry, such as LA volume index and LV mass index. However, there were no significant associations after adjustment. In conflict with previous findings, our study did not show any significant association with LVEF in the uni- or multivariable analysis (17, 18, 47). Possibly, this might be due to insufficient power in our study. More extensive studies are advocated to clarify these discrepant findings.

### Limitations

Twenty-six percent of the eligible patients declined participation. The dropouts did not differ significantly from the participants in sex and age. We had no data on disease burden, which might be a potential source of bias. Our results are limited to elderly Europeans and subjects with manifest PAD. Other concomitant diseases or medications beyond those presented have not been recorded. Other conditions’ influence cannot be excluded, although probably minor. Data on metformin medication were unavailable from participant questionnaires and could not be adjusted for. This might affect the association with diabetes as metformin increases levels of GDF-15.

Other limitations include the cross-sectional design of the study, from which we cannot draw any causal conclusions. Further, we have not adjusted for multiple hypothesis testing, which can impact type 1 errors in this study. The influence of the freezing procedure before analysis on the GDF-15 levels cannot be excluded, but previous data indicate that GDF-15 is robust to several freeze-thaw cycles (48). Data on smoking habits, physical activity, medical history, and medication use were based on self-reported information, which might be a cause of informational bias. However, to minimize the risk of this type of bias, prevalent medical diagnoses of diabetes and previous CVD were confirmed from medical records.

### Clinical implications and future perspectives

GDF-15 is an established marker that has been well-validated with good prognostic ability among patients with CVD in several studies. This holds promise for its clinical implementation. However, there are challenges, as GDF-15 is expressed in all organs (49) and has demonstrated valuable prognostic information across various diseases (2–5). Thus, it has emerged as a biomarker that lacks specificity and poses challenges to its clinical utility for risk stratification, particularly concerning elevated plasma levels. To better understand the association between GDF-15 levels and clinical outcomes, we conducted this study. Despite these efforts, our results suggest that GDF-15, in line with its low specificity, is more strongly associated with general clinical risk factors, especially diabetes and LDL cholesterol levels, than with specific indicators of atherosclerotic burden or cardiac dysfunction. Therefore, future investigations aimed at further elucidating the role of GDF-15 should primarily focus on the metabolic processes associated with atherosclerotic disease. However, the inherent sensitivity of GDF-15 could potentially serve as a valuable tool for identifying individuals at low risk for dismal prognosis. Therefore, it may contribute significantly to optimizing hospital resource allocation by enabling early discharge for such patients and establishing appropriate follow-up protocols within the realm of primary care. To validate the effectiveness of GDF-15 in guiding such management decisions, further prospective studies, preferably randomized studies, are imperative.

## Conclusion

Plasma levels of GDF-15 are more closely linked to traditional CV risk factors, such as diabetes, LDL cholesterol, and physical activity, than to direct indicators of atherosclerotic burden or cardiac dysfunction. To deepen understanding pathophysiological role of GDF-15 and its impact on clinical outcomes in patients with PAD, future studies should explore the metabolic pathways contributing to atherosclerotic disease.

## Supplementary Material


