# Supplementary material for: Determinants of growth differentiation factor 15 plasma levels in outpatients with peripheral arterial disease
Source: Ups J Med Sci. 2024 Dec 23;129:10.48101/ujms.v129.11001. doi: 10.48101/ujms.v129.11001 (PMC11708457; doi:10.48101/ujms.v129.11001)
Supplement: Supplementary file 1 [file UJMS-129-11001-s1.pdf]

Supplementary Material

Determinants of growth differentiation factor 15 plasma levels in outpatients with peripheral arterial disease

Emma Skau, Philippe Wagner, Jerzy Leppert, Johan Ärnlöv, and Pär Hedberg

Supplementary Figure 1. Density plots showing the distribution of GDF-15 according to subgroups of the study population. Continuous variables were dichotomised based on the median. The x-axis represents a logarithmic scale. Abbreviation: MI, myocardial infarction

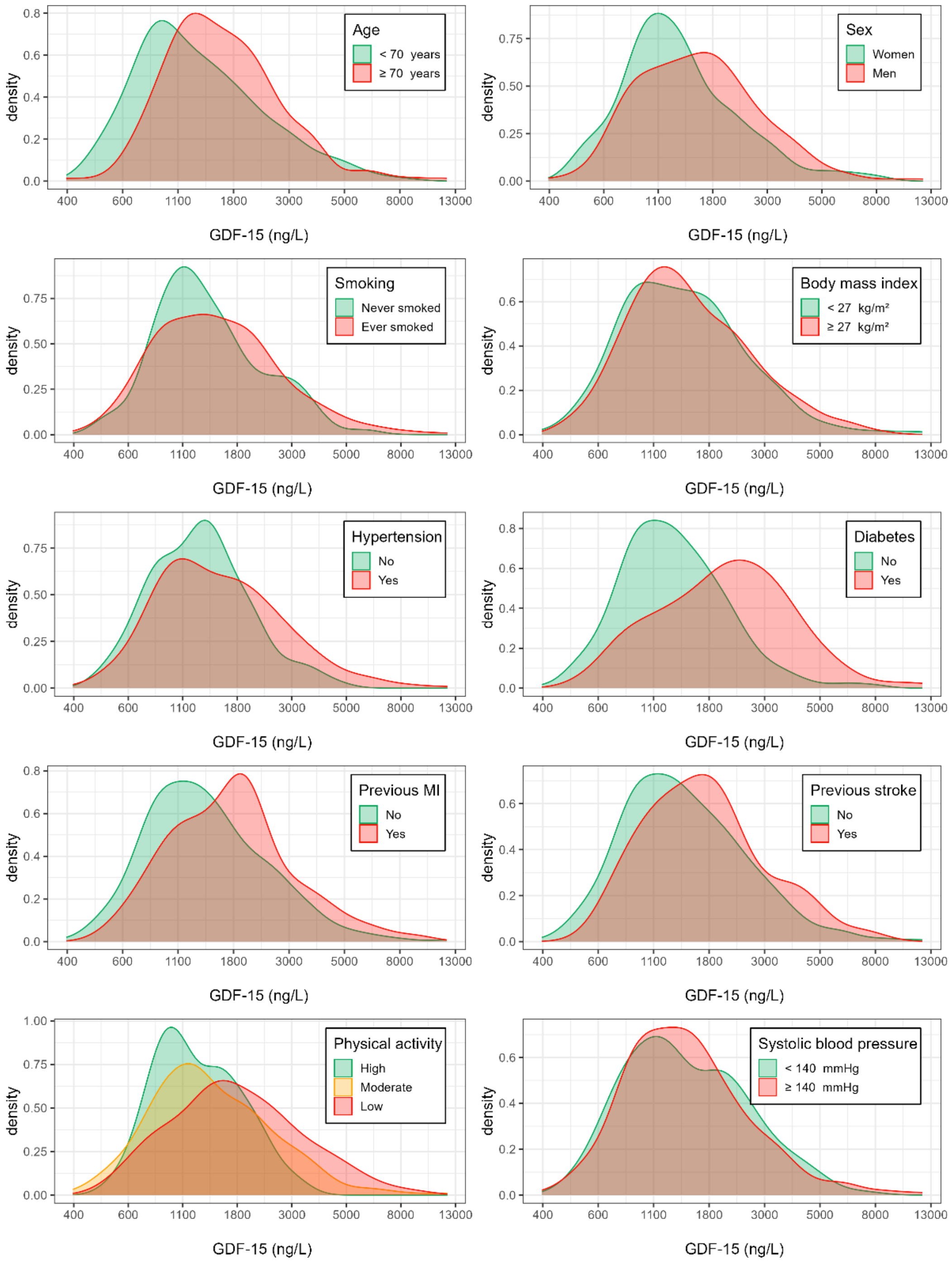

Supplementary Figure 2. Density plots showing the distribution of GDF-15 according to subgroups of the study population. Continuous variables were dichotomised based on the median. The x-axis represents a logarithmic scale.

Abbreviations: ABI, ancle brachial index; CRP, c-reactive protein eGFR, estimated glomerular filtration rate; ICA, internal carotid artery; LAV, left atrial volume; LDL, low density lipoprotein; LV, left ventricular; LVEF, left ventricular ejection fraction.

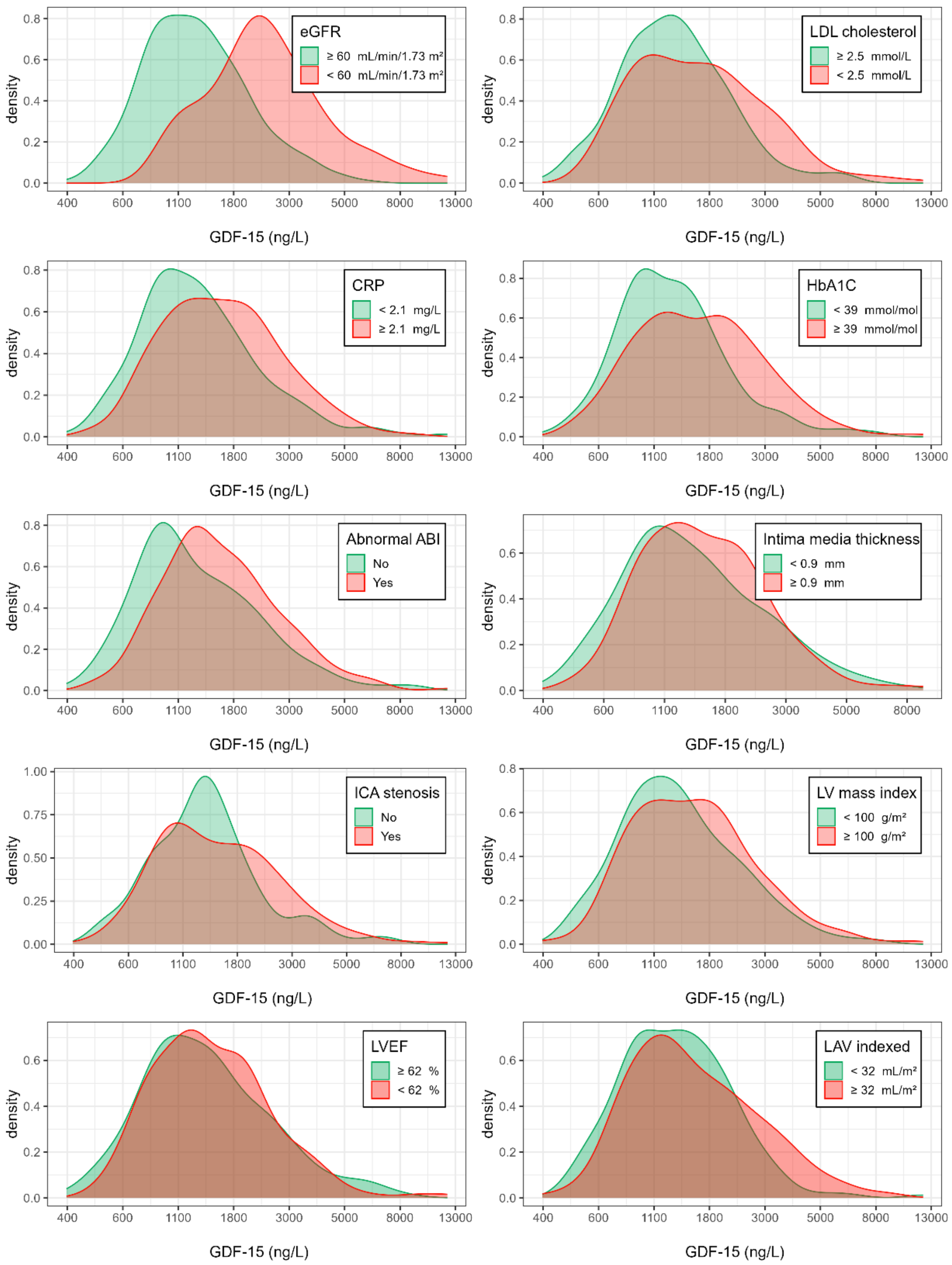

Supplementary Figure 3. Scatter plot showing the nonlinear correlation between the natural logarithm of growth differentiation factor 15 (GDF-15) and estimated glomerular filtration rate (eGFR).

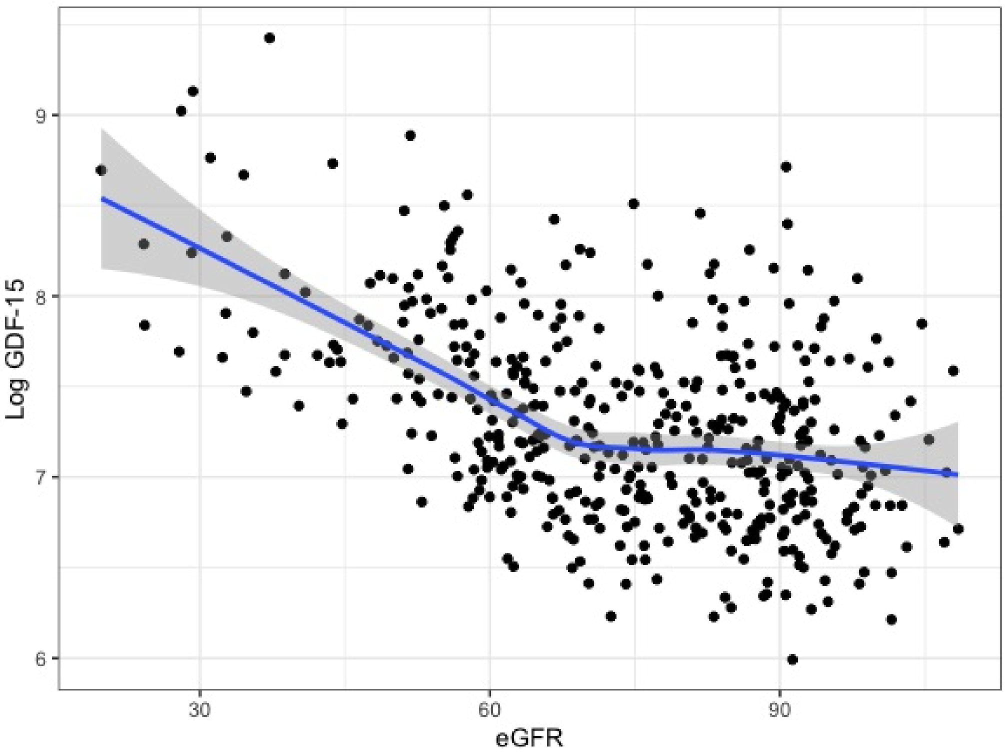

Supplementary Figure 4. Scatter plot showing the nonlinear correlation between the natural logarithm of growth differentiation factor 15 (GDF-15) and low-density lipoprotein (LDL) cholesterol.

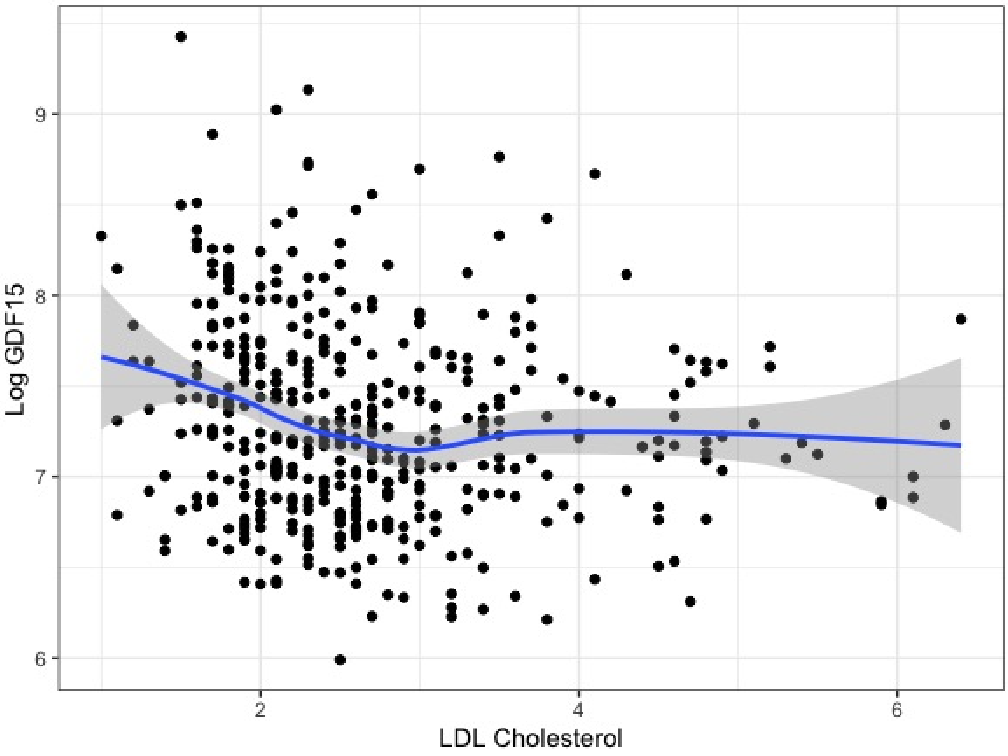

Supplementary Figure 5. Forest plot showing associations between the natural logarithm of GDF-15 plasma levels and various clinical parameters in the complete case dataset (i.e. patients with missing values were excluded). The estimates are beta coefficients and 95% CI for every standard deviation increase in continuous variables and presence versus absence for categorical variables. For every variable, the first coefficient was unadjusted and the second was adjusted for the other variables.

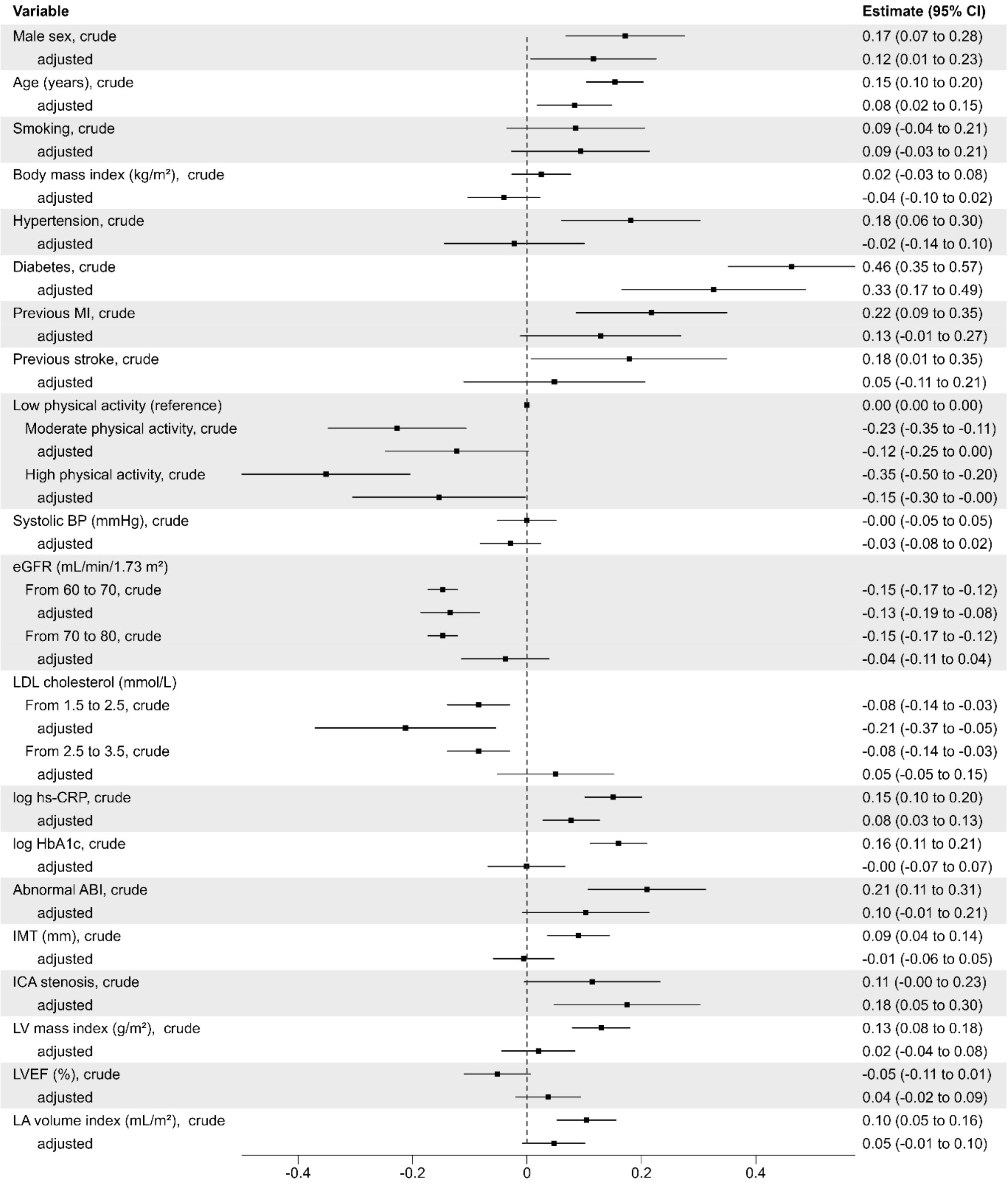

ABI, ankle-brachial index; BMI, body mass index; BP, blood pressure; CI, confidence interval; CV, cardiovascular; eGFR, estimated glomerular filtration rate; GDF-15, growth differentiation factor 15; HbA1c, glycated haemoglobin; hs-CRP, high-sensitive C-reaction protein; ICA, internal carotid artery; IMT, intima-media thickness; LA, left atrial; LDL, low-density-lipoprotein cholesterol; LV, left ventricular; LVEF, LV ejection fraction; MI, myocardial infarction.

Supplementary Figure 6. Forest plot showing the associations between the natural logarithm of GDF-15 plasma levels and various clinical parameters. The estimates are beta coefficients and 95% CI for every standard deviation increase in continuous variables and presence versus absence for categorical variables. For every variable, the first coefficient was unadjusted and the second was adjusted for the other variables. The results were pooled from 50 multiply imputed datasets, where the imputed values for the left ventricular ejection fraction were decreased by 20%.

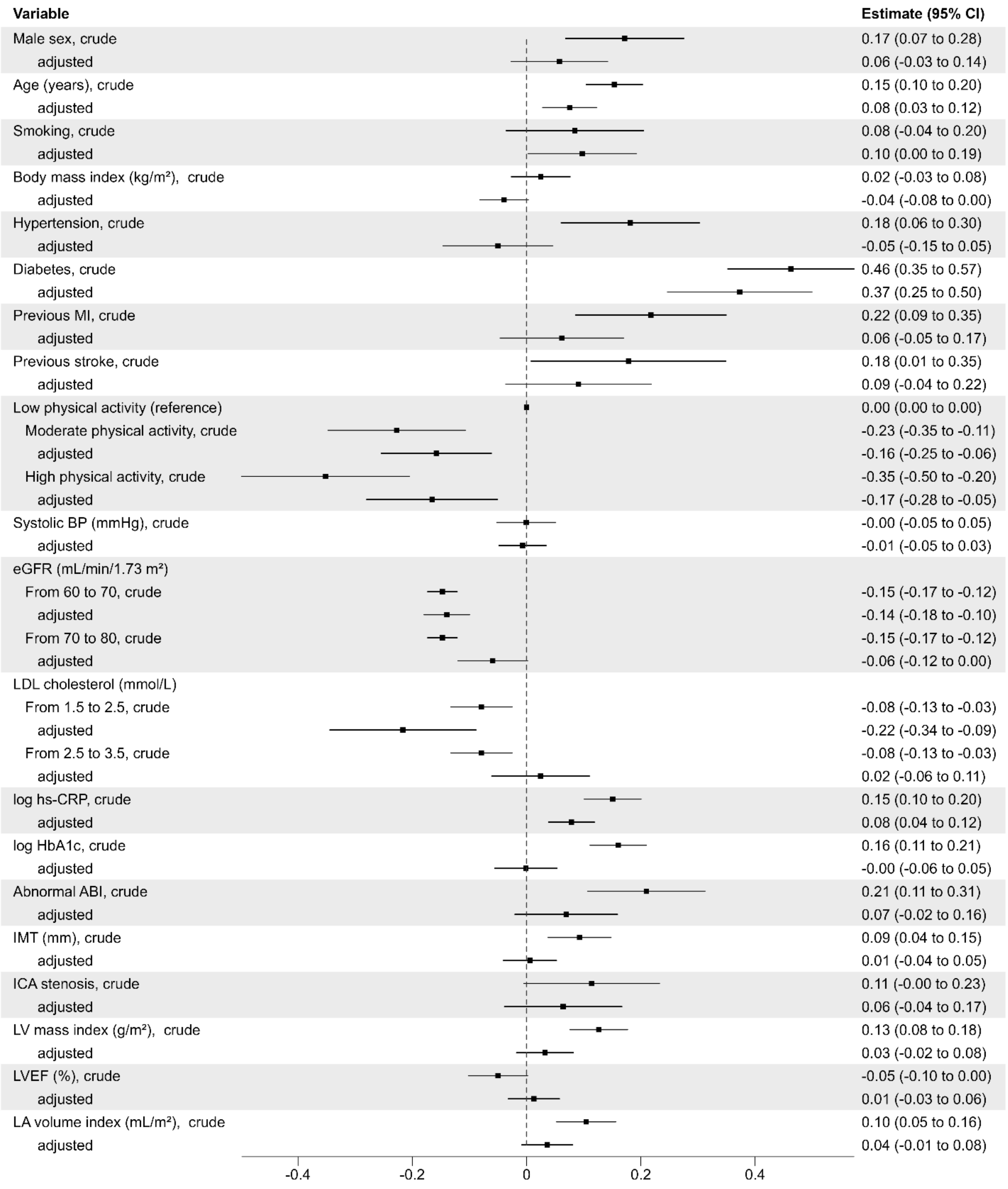

Abbreviations are the same as in Supplementary Figure 3.

Supplementary Figure 7. Forest plot showing the associations between the natural logarithm of GDF-15 plasma levels and various clinical parameters. The estimates are beta coefficients and 95% CI for every standard deviation increase in continuous variables and presence versus absence for categorical variables. For every variable, the first coefficient was unadjusted and the second was adjusted for the other variables. The results were pooled from 50 multiply imputed datasets, where the imputed values for the left ventricular ejection fraction were increased by 20%.

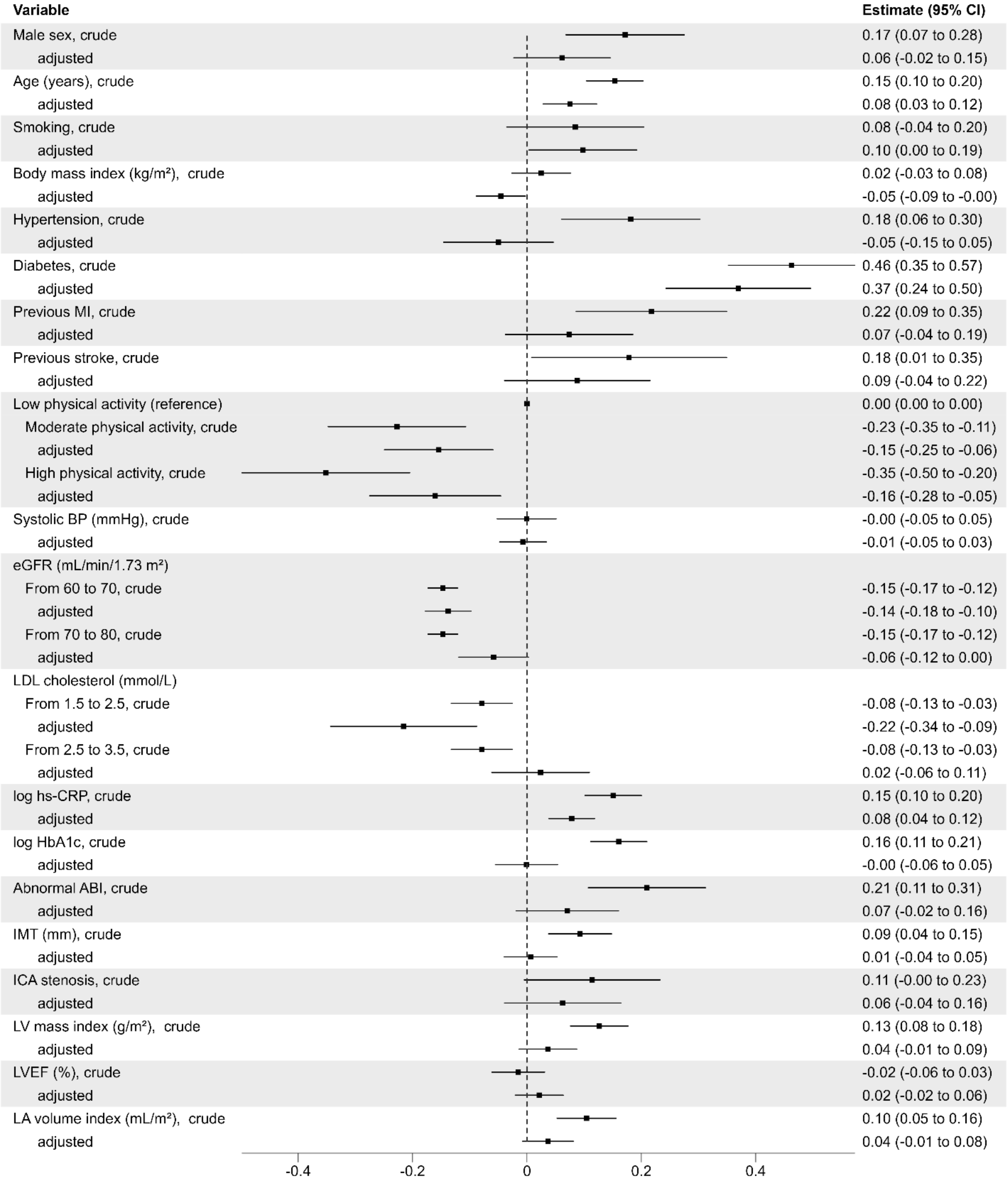

Abbreviations are the same as in Supplementary Figure 3.

Supplementary Figure 8. Forest plot showing the associations between the natural logarithm of GDF-15 plasma levels and various clinical parameters. The estimates are beta coefficients and 95% CI for every standard deviation increase in continuous variables and presence versus absence for categorical variables. For every variable, the first coefficient was unadjusted and the second was adjusted for the other variables. The results were pooled from 50 multiply imputed datasets, where the imputed values for intima-media thickness decreased by 20%.

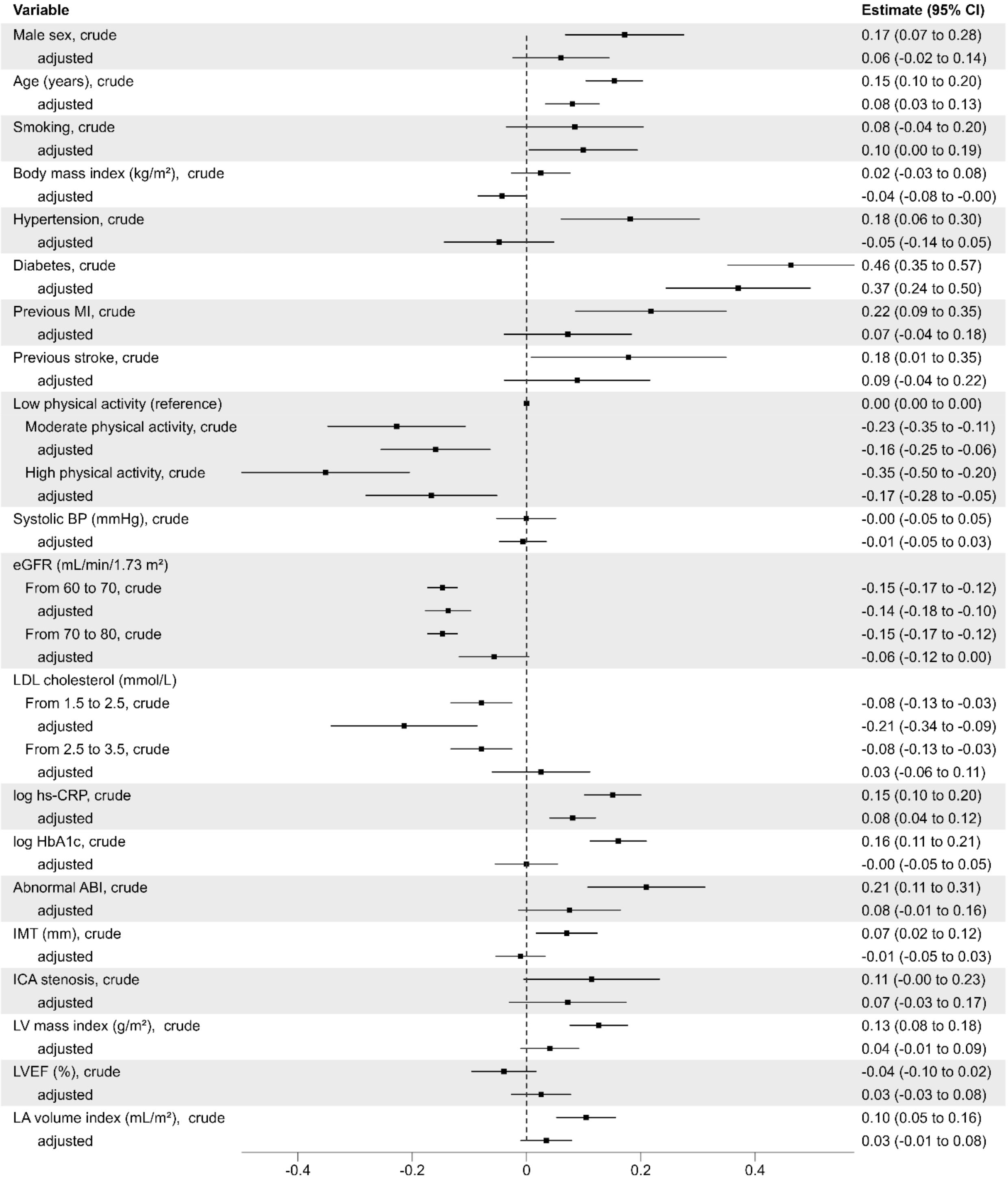

Abbreviations are the same as in Supplementary Figure 3.

Supplementary Figure 9. Forest plot showing the associations between the natural logarithm of GDF-15 plasma levels and various clinical parameters. The estimates are beta coefficients and 95% CI for every standard deviation increase in continuous variables and presence versus absence for categorical variables. For every variable, the first coefficient was unadjusted and the second was adjusted for the other variables. The results were pooled from 50 multiply imputed datasets, where the imputed values for intima-media thickness were increased by 20%.

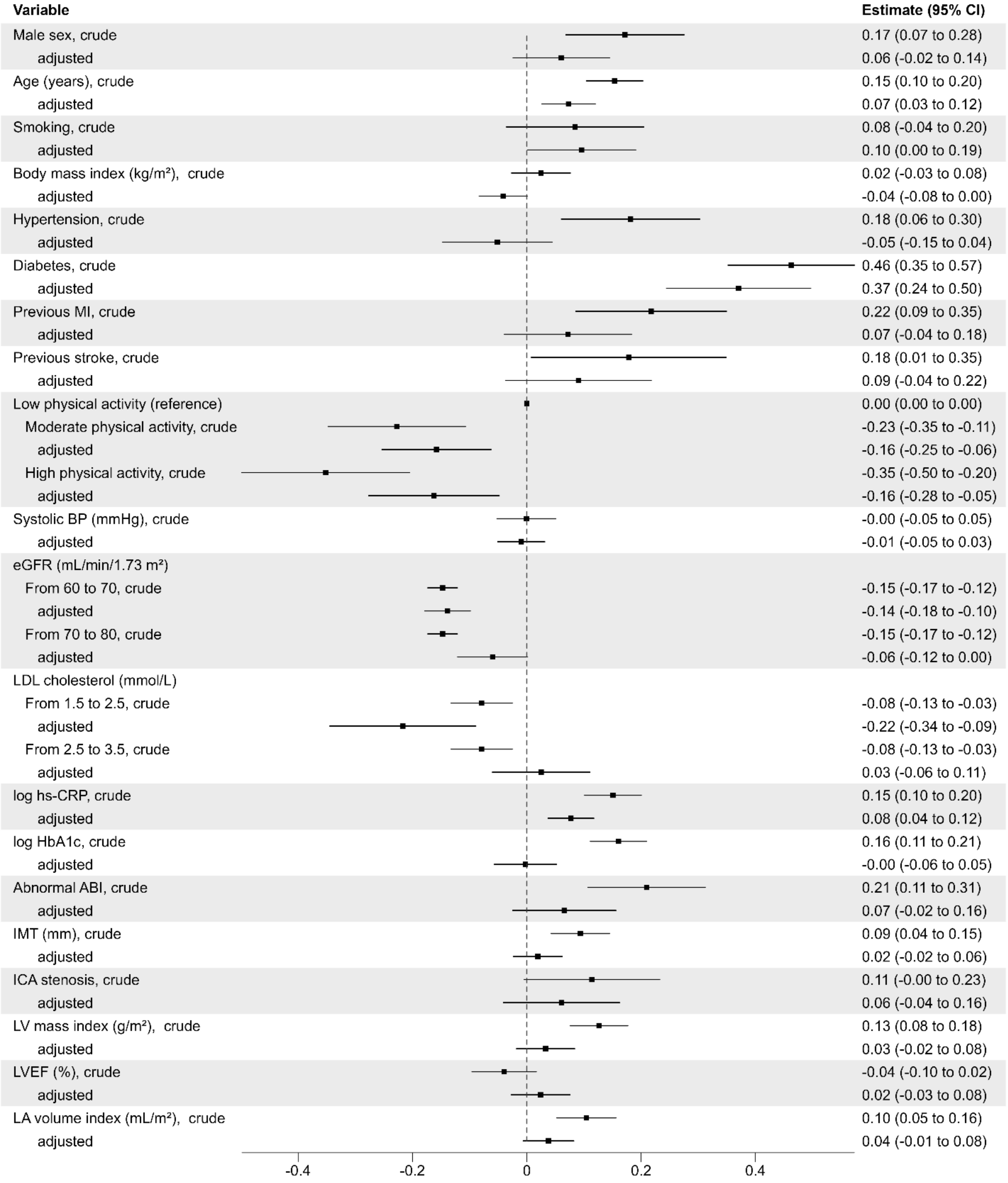

Abbreviations are the same as in Supplementary Figure 3.
